# Exposure to a Predator Scent Induces Chronic Behavioral Changes in Rats Previously Exposed to Low-level Blast: Implications for the Relationship of Blast-Related TBI to PTSD

**DOI:** 10.3389/fneur.2016.00176

**Published:** 2016-10-18

**Authors:** Georgina Perez-Garcia, Miguel A. Gama Sosa, Rita De Gasperi, Margaret Lashof-Sullivan, Eric Maudlin-Jeronimo, James R. Stone, Fatemeh Haghighi, Stephen T. Ahlers, Gregory A. Elder

**Affiliations:** ^1^Department of Neurology, Icahn School of Medicine at Mount Sinai, New York, NY, USA; ^2^Research and Development Service, James J. Peters Department of Veterans Affairs Medical Center, Bronx, NY, USA; ^3^Friedman Brain Institute, Icahn School of Medicine at Mount Sinai, New York, NY, USA; ^4^Department of Psychiatry, Icahn School of Medicine at Mount Sinai, New York, NY, USA; ^5^Department of Neurotrauma, Operational and Undersea Medicine, Naval Medical Research Center, Silver Spring, MD, USA; ^6^Department of Radiology and Medical Imaging, University of Virginia, Charlottesville, VA, USA; ^7^Department of Neurosurgery, University of Virginia, Charlottesville, VA, USA; ^8^Fishberg Department of Neuroscience, Icahn School of Medicine at Mount Sinai, New York, NY, USA; ^9^Neurology Service, James J. Peters Department of Veterans Affairs Medical Center, Bronx, NY, USA

**Keywords:** animal models, blast, postconcussion syndrome, post-traumatic stress disorder, rat, traumatic brain injury

## Abstract

Blast-related mild traumatic brain injury (mTBI) has been unfortunately common in veterans who served in the recent conflicts in Iraq and Afghanistan. The postconcussion syndrome associated with these mTBIs has frequently appeared in combination with post-traumatic stress disorder (PTSD). The presence of PTSD has complicated diagnosis, since clinically, PTSD and the postconcussion syndrome of mTBI have many overlapping symptoms. In particular, establishing how much of the symptom complex can be attributed to the psychological trauma associated with PTSD in contrast to the physical injury of traumatic brain injury has proven difficult. Indeed, some have suggested that much of what is now being called blast-related postconcussion syndrome is better explained by PTSD. The relationship between the postconcussion syndrome of mTBI and PTSD is complex. Association of the two disorders might be viewed as additive effects of independent psychological and physical traumas suffered in a war zone. However, we previously found that rats exposed to repetitive low-level blast exposure in the absence of a psychological stressor developed a variety of anxiety and PTSD-related behavioral traits that were present months following the last blast exposure. Here, we show that a single predator scent challenge delivered 8 months after the last blast exposure induces chronic anxiety related changes in blast-exposed rats that are still present 45 days later. These observations suggest that in addition to independently inducing PTSD-related traits, blast exposure sensitizes the brain to react abnormally to a subsequent psychological stressor. These studies have implications for conceptualizing the relationship between blast-related mTBI and PTSD and suggest that blast-related mTBI in humans may predispose to the later development of PTSD in reaction to subsequent psychological stressors.

## Introduction

Blast-related mild traumatic brain injuries (mTBI) were common in the recent conflicts in Iraq and Afghanistan. One of the striking features of the postconcussion syndromes associated with these mTBIs has been the concomitant presence of post-traumatic stress disorder (PTSD) (1). Initial reports indicated that PTSD or depression were present in over one-third of veterans who had recently returned from Iraq and were suspected of suffering from mTBI-related postconcussion symptoms (2). Many later studies confirmed these findings, and among veterans with ongoing symptoms thought to be attributable to blast-related mTBI, most have PTSD (1).

However, the relationship between the postconcussion syndrome of mTBI and PTSD is complex. The presence of PTSD complicates diagnosis since clinically distinguishing the postconcussion syndrome from PTSD is often difficult due to the overlapping symptoms of the two disorders (1). In both, a combination of somatic, affective, and cognitive complaints is present. Results of neuropsychological testing are similar with altered attention and concentration as well as mild deficits in memory and executive function present in both (3). The diagnostic distinction has been further complicated by the lowering of the threshold for labeling the “event” needed to establish a traumatic brain injury (TBI) diagnosis to the most transient alterations of consciousness (1). This has led to far more events being labeled as “TBIs” suggesting to some that blast-induced mTBI is now being overdiagnosed (4) with many of the symptoms being attributed to a blast-related postconcussion syndrome better explained by PTSD (1). Indeed, a 2014 Institute of Medicine report (5) on the long-term consequences of blast injury, while acknowledging the limited nature of evidence in humans, nevertheless concluded that there is “limited/suggestive evidence that most of the shared symptoms are accounted for by PTSD and not a direct result of TBI alone.” Thus, skepticism exists concerning the nature of the blast-related mTBI/PTSD complex.

Many animal models use physical stressors (such as inescapable footshocks) or psychogenic stressors (such as predator scent) to induce behavioral traits in animals that resemble the symptoms associated with human PTSD (6). The predator scent challenge has been successfully used to induce PTSD-like behavior as well as to assess anxiety status in rodents (7). Previously, we described an animal model of blast exposure in which rats were subjected to three low-level exposures delivered one blast exposure per day on three consecutive days (8). Blast-exposed rats developed a variety of chronic anxiety and PTSD-related behavioral traits (9). Blast-exposed animals also exhibited an altered response to a predator scent challenge after exposure (9). Here, we show that a single predator scent challenge delivered 8 months after the last blast exposure induces chronic behavioral changes that are still present 45 days later. These observations have implications for understanding the relationship of blast-related TBI to PTSD.

## Materials and Methods

### Animals

Adult male Long Evans Hooded rats (250–350 g; 10 weeks of age; Charles River Laboratories International, Inc., Wilmington, MA, USA) were used as subjects. All studies were reviewed and approved by the animal care and use committees of the Walter Reed Institute of Research/Naval Medical Research Center and the James J. Peters VA Medical Center and were conducted in compliance with the Public Health Service policy on the humane care and use of laboratory animals, the NIH Guide for the Care and Use of Laboratory Animals, and all applicable Federal regulations governing the protection of animals in research.

### Blast Overpressure Exposure

Rats were exposed to overpressure injury using the Walter Reed Army Institute of Research (WRAIR) shock tube, which simulates the effects of air blast exposure under experimental conditions. The shock tube has a 12″ circular diameter and is a 19.5-ft long steel tube divided into a 2.5-ft compression chamber that is separated from a 17-ft expansion chamber. The compression and expansion chambers are separated by polyethylene Mylar™ sheets (Du Pont Co., Wilmington, DE, USA) that control the peak pressure generated. The peak pressure at the end of the expansion chamber was determined by piezoresistive gages specifically designed for pressure–time (impulse) measurements (Model 102M152, PCB, Piezotronics, Inc., Depew, NY, USA). This apparatus has been used in multiple prior studies to deliver blast overpressure injury to rats (8–16).

Individual rats were anesthetized using an isoflurane gas anesthesia system consisting of a vaporizer, gas lines, and valves and an activated charcoal scavenging system adapted for use with rodents. Rats were placed into a polycarbonate induction chamber, which was closed and immediately flushed with 5% isoflurane in air mixture for 2 min. Rats were placed into a cone shaped plastic restraint device and then placed in the shock tube. Movement was further restricted during the blast exposure using 1.5-cm diameter flattened rubber tourniquet tubing. Three tourniquets were spaced evenly to secure the head region, the upper torso, and lower torso while the animal was in the plastic restraint cone. The end of each tubing was threaded through a toggle and run outside of the exposure cage where it was tied to firmly affix the animal and prevent movement during the blast overpressure exposure without restricting breathing. Rats were randomly assigned to sham or blast conditions and were placed in the shock tube lying prone with the plane representing a line from the tail to the nose of the body in line with the longitudinal axis of the shock tube with the head placed more upstream. Further details of the physical characteristics of the blast wave are described in Ahlers et al. (8). Blast-exposed animals received 74.5 kPa (duration 4.8 ms, impulse 175.8 kPa × ms) exposures administered one exposure per day for three consecutive days. Sham-exposed animals were treated identically including receiving anesthesia and being placed in the blast tube but did not receive a blast exposure. Subjects received blast overpressure exposure at the Naval Medical Research Center, and the day following the last blast exposure, they were transferred to the James J. Peters VA Medical Center where all other procedures were performed.

### Animal Housing for Behavioral Testing

Animals were housed at a constant 70–72°F temperature with rooms on a 12:12-h light cycle with lights on at 07:00 a.m. All subjects were individually housed in standard clear plastic cages equipped with Bed-O’Cobs laboratory animal bedding (The Andersons, Maumee, OH, USA) and EnviroDri nesting paper (Sheppard Specialty Papers, Milford, NJ, USA). Access to food and water was *ad libitum*. Subjects were housed on racks in random order to prevent rack position effects. All behavioral testing was performed by the same investigator (Georgina Perez-Garcia).

### Predator Scent Exposure

Before exposure, baseline activity of subjects was measured in an open field chamber for 10 min. Twenty ml of cat urine (Research Inc., Waverly, NY, USA) was mixed with 100 ml of bedding (standard corn husk) and shaken together in a flask before being spread evenly around the bottom of an identical open field chamber. Subjects were placed in the chamber for 10 min, and activity was recorded during the period of exposure. Following exposure, subjects were transferred to a clean open field cage, and activity was recorded for an additional 40 min. At 3 days postexposure, open field activity was recorded for 30 min, and at 45 days postexposure, it was recorded for 60 min. Open field activity was determined as previously described (9).

### Statistical Analysis

Statistical tests employed unpaired *t-*tests or repeated measures analysis of variance (ANOVA). Equality of variance was assessed using the Levene test. When the Levene test was not significant (*p* > 0.05) between groups, comparisons were made using Student’s *t*-test. If the Levene statistic was significant (*p* < 0.05), unpaired *t-*tests were employed using the Welch correction for unequal variances. When repeated measures ANOVA was used, sphericity was assessed using Mauchly’s test. If the assumption of sphericity was violated (*p* < 0.05, Mauchly’s test), significance was determined using the Greenhouse–Geisser correction. Statistical tests were performed using the program GraphPad Prism 7.0 (GraphPad Software, San Diego, CA, USA) or SPSS 23.0 (SPSS, Chicago, IL, USA).

## Results

### A Predator Scent Challenge in Blast-Exposed Rats Induces a Sustained Behavioral Response

**Figure 1:**
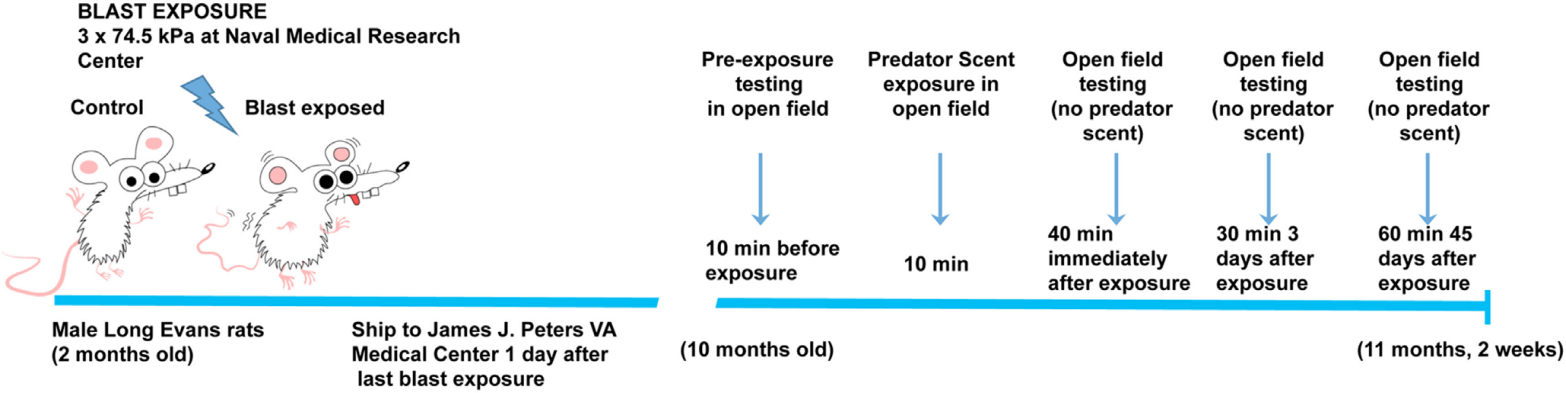
**Time line of blast injury, predator scent exposure, and open field-testing**. Blast exposure occurred at the Naval Medical Research Center when animals were 2 months of age. Animals were shipped to the James J. Peters VA Medical Center the day following the last blast exposure. Predator scent exposure occurred 8 months later when rats were 10 months old. Prior to predator scent exposure, subjects were placed in an open field chamber for 10 min, and activity was recorded. Rats were then transferred to an open field chamber containing cage bedding that had been soaked in cat urine. Activity was recorded during the 10 min of exposure. Rats were then transferred to a clean open field cage (no predator scent present), and activity was recorded for an additional 40 min. At 3 days postexposure, open field activity was recorded for 30 min (no predator scent present), and at 45 days postexposure, it was recorded for 60 min (no predator scent present).

**Figure 2:**
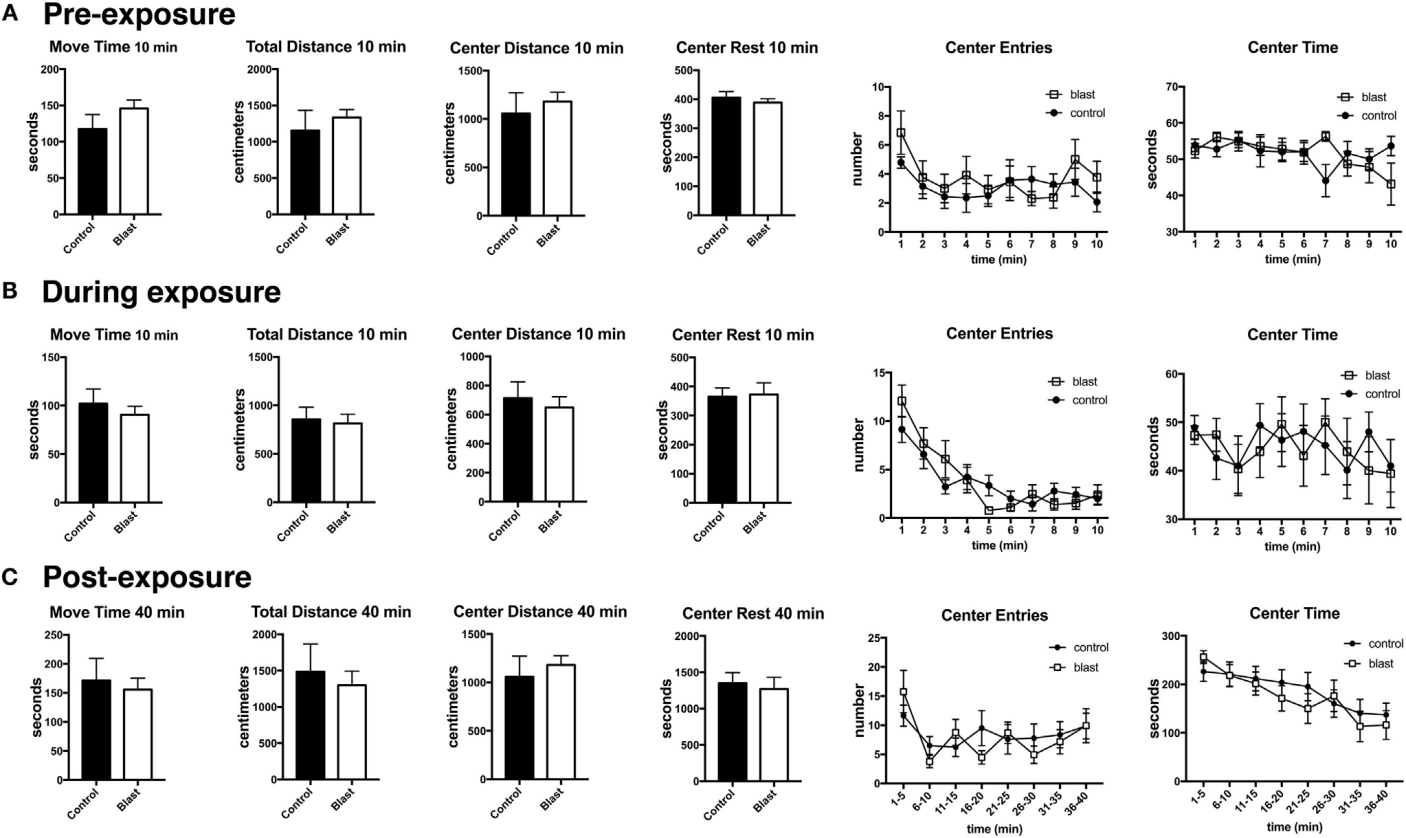
**No acute changes in open field activity of blast-exposed rats during or immediately following a predator scent exposure**. Open field activity (move time, total distance, center distance, center rest time, center entries, and center time) was measured before **(A)**, during **(B)**, and immediately after **(C)** exposure to a predator scent. Error bars indicate ±SEM. There were no statistically significant differences between blast-exposed and controls in any of the measures (unpaired *t*-tests). See text for discussion of additional statistical testing.

Previously, we found that blast exposure induced an altered response to a predator scent challenge during and immediately after the predator scent exposure (9). Here, we administered a similar predator scent challenge to an additional group of rats 8 months following blast exposure. The outline of the studies is shown in Figure 1. Initially, we measured baseline activity in an open field. Rats were next transferred to an open field chamber containing cage bedding that was soaked in cat urine. Activity was recorded during the 10 min of exposure. Rats were next transferred to a clean open field cage, and activity was recorded for another 40 min. While center entries decreased with open field time before (repeated measures ANOVA, *F*_5.5,138.8_ = 2.60, *p* = 0.023), during (*F*_5.4,134.0_ = 17.5, *p* < 0.0001), and after (*F*_4.8,120.5_ = 4.48, *p* = 0.001) exposure, there were no differences between blast-exposed and controls in center entries before (freezing × condition, *F*_5.5,138.8_ = 0.927, *p* = 0.47), during (freezing × condition, *F*_5.4,134.0_ = 1.62, *p* = 0.15), or in the immediate (freezing × condition, *F*_4.8,120.5_ = 4.48, *p* = 0.19) postexposure periods (Figure 2). Indeed, no differences were found between blast-exposed and controls in any parameter measured in the open field before, during, or in the immediate postexposure period (Figure 2).

**Figure 3:**
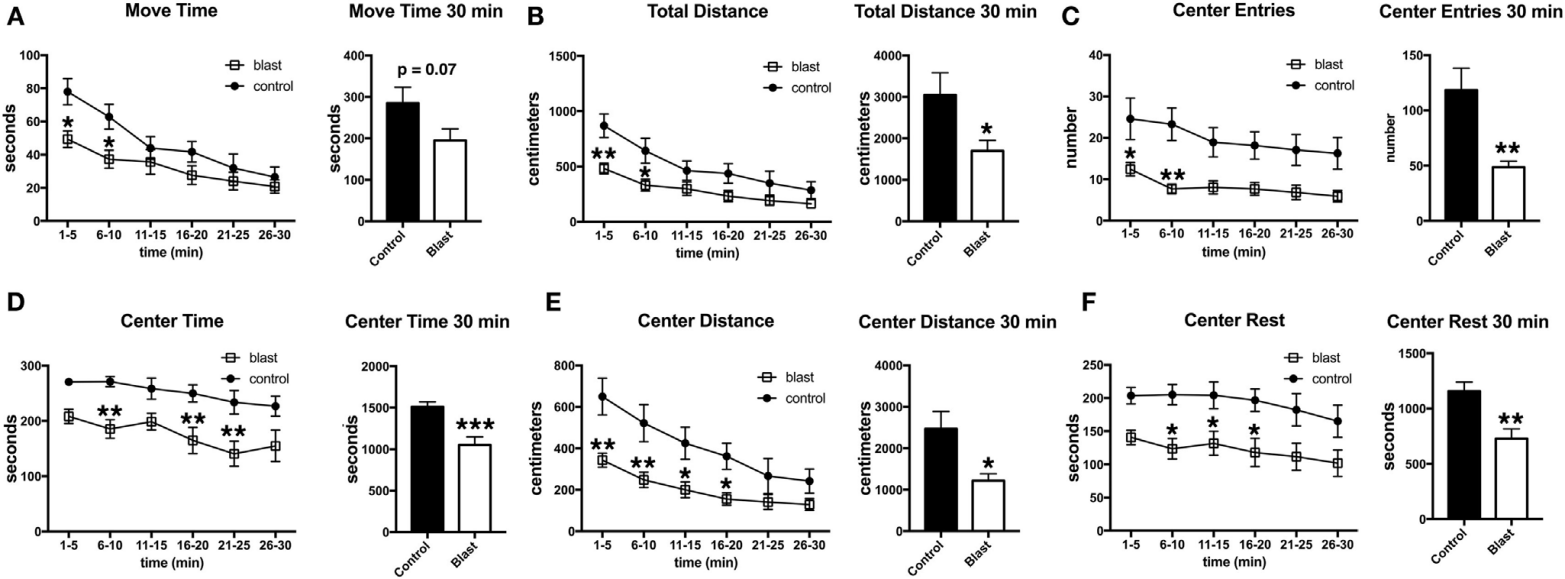
**Delayed behavioral changes in blast-exposed rats at 3 days following a single predator scent exposure**. Activity of blast-exposed and control rats was assessed in an open field for 30 min at 3 days after the predator scent exposure. Shown are time in motion **(A)**, total distance moved **(B)**, center entries **(C)**, center time **(D)**, distance traveled in the center **(E)**, and center rest time **(F)**. Error bars indicate ±SEM. Asterisk(s) indicate statistically significant differences between blast-exposed and control (*p < 0.05, **p < 0.01, unpaired *t*-tests).

Three days following the predator scent exposure, open field activity was recorded for 30 min. No predator scent was present during this testing. Despite the lack of differences between blast-exposed and controls in activity during the acute and immediate postexposure period (Figure 2), at 3 days after exposure, blast-exposed rats spent less time in motion and moved less in the open field (Figure 3). Blast-exposed animals also made fewer center entries as well as traveled less distance in the center of the field and exhibited a decreased center rest time (Figure 3).

**Figure 4:**
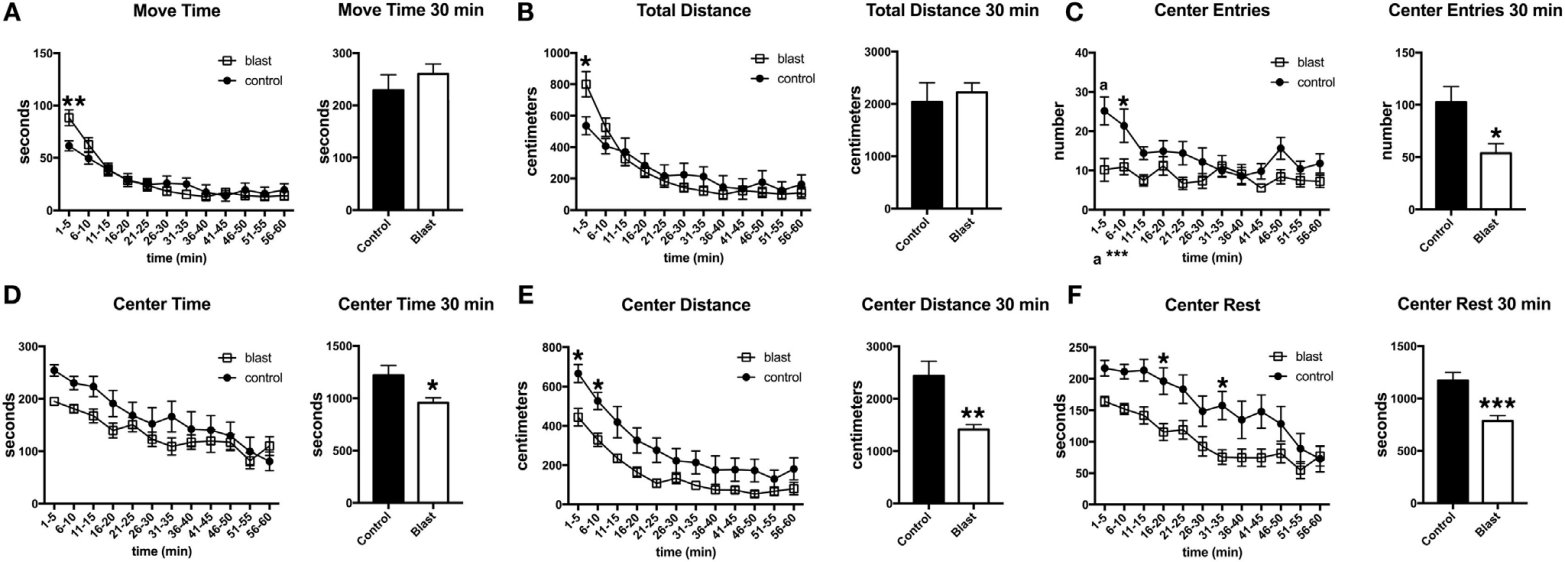
**Behavioral changes are still present 45 days following a predator scent exposure**. Activity of blast and control rats was assessed in an open field for 60 min at 45 days after the predator scent exposure. Shown are time in motion **(A)**, total distance moved **(B)**, center entries **(C)**, center time **(D)**, distance traveled in the center **(E)**, and center rest time **(F)**. Error bars indicate ±SEM. Asterisk(s) indicate statistically significant differences between blast-exposed and control [*p < 0.05, **p < 0.01, ***p < 0.001, a in **(C)** indicates *p* < 0.001, all comparisons unpaired *t*-tests].

To determine whether the response was sustained, open field activity was recorded for 60 min at 45 days postexposure in the absence of a predator scent (Figure 4). As at 3 days postexposure, blast-exposed rats made fewer center entries, traveled less in the center of the open field, and exhibited a decreased center rest time. Thus, a single predator scent exposure induces persistent short- and long-term behavioral effects in blast-exposed rats that are still present 45 days after the initial predator scent exposure.

## Discussion

Traumatic brain injury and PTSD are complex disorders with historical links dating back to the entity known as “shell shock,” which was first recognized during World War I. As part of the static trench warfare, which was characteristic of this era, British troops were exposed to a variety of blasts at close range (17). Symptoms developed that had features of the postconcussion syndrome as well as what would now be called PTSD. A debate as to whether shell shock resulted from physical injury or psychic trauma ended without any clear resolution (17). Echoes of this argument linger on in the current debate over the causes of the blast-related mTBI/PTSD complex in the most recent veterans returning from Iraq and Afghanistan (1).

Traumatic brain injury and PTSD can be viewed as different ends of a spectrum, TBI representing the prototypical example of an organic brain disease caused by direct physical trauma. By contrast, PTSD is regarded as a psychologically based reaction to a stressor that did not cause physical injury. Indeed, it has even been suggested that the post-traumatic amnesia associated with TBI may serve as a protection against PTSD later developing (18), and although PTSD can be seen after even moderate to severe TBI, PTSD does appear to occur less frequently in subjects with no memory for the event (19, 20).

Studies using animals should permit effects of the primary blast wave to be determined free of many of the confounding variables present in natural human exposures. Animals have been exposed to various forms of blast ranging from direct exposure to live explosives to more commonly controlled blast waves produced by compressed-air generators in shock tubes (1, 21). However, blast overpressure dynamics in the field is complex. Under conditions where environmental structures permit reflection of the blast wave, a perfect Friedlander wave is typically not observed (22, 23). Shock tubes offer more experimental control permitting the effects of blast overpressure to be studied in isolation. However, the overpressure wave produced by conventional shock tubes typically differs from that of native explosives (24), and in the laboratory, neither live explosives nor shock tubes readily replicate the non-ideal blast wave with its multiple shock and expansion fronts that occur in real-life settings.

The current studies were performed using the shock tube at the WRAIR. This shock tube has been used in multiple prior studies to deliver blast overpressure injury to rats (8–16). The blast wave overpressure profile used in these studies has been demonstrated to produce mild neurological impairments in rats acutely (8). These exposures are within a peak pressure range where neurological disruption has been observed in training exercises involving human breachers (25). The nose cone and physical restraints used in the present studies to limit head and body motion may also affect the overpressure experienced by the subject. Although the blast exposures experienced by the animals in this study are somewhat representative of exposures witnessed by breachers, they may not fully replicate the exposures witnessed by breachers or soldiers in theater.

How blast damages the brain remains incompletely understood although a substantial body of data supports a role for vascular and inflammatory factors as being key (26). If blast’s regional anatomic effects damage brain structures that are critical in the development of PTSD, then blast-injured rats might exhibit an increased sensitivity to PTSD-related stressors. Biological models of PTSD postulate that alterations in key frontal and limbic structures, including the prefrontal cortex, amygdala, and hippocampus, are involved in the development of PTSD (27, 28). Based largely on human neuroimaging data, these models suggest that inadequate frontal inhibition of the amygdala leads to exaggerated amygdala responses to psychological threats. Supporting neuroanatomically based theories of PTSD, a study in brain-injured Vietnam War veterans found that damage to the ventromedial prefrontal cortex and amygdala was associated with a reduced occurrence of PTSD (29). In rats, previous work has shown that in addition to inducing PTSD-related behaviors such as anxiety, exposure to a predator odor (urine or cat fur odor) increases GluN1 protein expression in the amygdala and hippocampus (30). Predator scent exposure in rats also causes changes in the extent, distribution, and morphology of dendrites in the hippocampus and amygdala (31) as well as alters levels of gonadal steroid hormones (32).

Explaining the basis for the chronic symptoms in dual diagnosis veterans has been challenging. In particular, establishing how much of the mTBI/PTSD symptom complex can be attributed to the psychological trauma associated with PTSD as opposed to the physical injury of TBI has proven difficult. Association of the two disorders in veterans returning from a war zone could easily be explained by exposures to PTSD-related stressors independent of TBI. In this model, the apparent mTBI/PTSD complex could be viewed as the additive results of independent psychological and physical traumas.

A small number of studies have begun to address dual exposures to TBI and psychological stressors in animal models. Klemenhagen et al. (33) found synergistic effects when a repetitive closed impact TBI injury was combined with a foot shock, observing greater effects on impairments in social recognition and depression-like behavior with the dual exposure. However, Ojo et al. (34) found that when a controlled cortical impact injury was combined with a predator scent and inescapable foot shock exposure, the combined insult actually abrogated effects on contextual fear and impairments in social behavior. Behavioral traits may also be affected when blast is combined with repeated stress (35) or factors, such as transportation or anesthesia (36).

The findings presented here suggest a relationship more complex than a simple additive model. We previously found that when young adult male rats were subjected to three low-level blast exposures, PTSD-related behavioral traits developed that could be seen many months following the last blast exposure (9). Here, rats subjected to similar blast exposures were presented with a predator scent challenge 8 months later. On the day of predator scent exposure, no behavioral changes were seen in the open field activity either during or immediately after the predator scent challenge. However, 3 days later, a clear anxiety response was seen in blast-exposed animals, which was still present 45 days following the initial exposure.

These results differ slightly from our previous results when a predator scent exposure was administered 6 months after the last blast exposure (9). In those studies, blast-exposed rats exhibited an anxiety response during and immediately after the predator scent exposure. However, the predator scent-induced effect in blast-exposed animals appeared stronger 3 days later (9). The present studies confirm the altered responsiveness of previously blast-exposed rats to a predator scent and extend those earlier studies (9) by showing that the effect is still present 45 days following the predator scent exposure. Collectively, these observations suggest that a single predator scent exposure administered long after blast injury can cause a sustained change in behavior.

Supporting heightened amygdala function in blast-exposed rats, we previously observed increased cued responses in a fear-conditioning paradigm (9) associated with elevation of the protein stathmin 1 in the amygdala (9). Elevation of stathmin 1 was notable since it is highly expressed in the amygdala, and when stathmin 1 was knocked out in the mouse, the resulting animals were impaired in their ability to learn fear responses (37). Blast-exposed mice also exhibit altered fear responses and in the basolateral amygdala have fewer numbers of a subclass of projection neuron linked to fear suppression (38). Recently, pyramidal neurons in the amygdala of blast-exposed rats have been shown to exhibit increased dendritic arbors (39) further supporting the notion of heightened amygdala function.

Thus, the relationship between blast-related TBI and PTSD may not be completely explained by a simple additive model involving independent psychological and physical stressors. Prior studies in animals have shown that blast induces PTSD-related traits without the need to add a psychological stressor (9, 38). The studies presented here further suggest that blast exposure can sensitize the brain to react abnormally to a subsequent psychological stressor. These observations have implications for understanding the relationship of blast-related TBI to PTSD, implying a complex relationship and further suggesting that human blast-related mTBI may predispose to the later development of PTSD following a subsequent psychological stressor.

## Author Contributions

GP-G conducted the behavioral testing. GP-G and GE analyzed the behavioral data. ML-S, EM-J, and SA conducted the blast exposures. All authors (GP-G, MS, RG, ML-S, EM-J, JS, FH, SA, and GE) participated in the drafting and revising of the manuscript.

## Conflict of Interest Statement

The authors declare that this research was conducted in the absence of any commercial or financial relationships that could be construed as a potential conflict of interest.
